# Lightning Strike-Induced Myocarditis

**DOI:** 10.7759/cureus.32443

**Published:** 2022-12-12

**Authors:** Ojas A Mahajan, Satish Mahajan, Vivek Lahane, Nitish Batra

**Affiliations:** 1 Department of Medicine, Jawaharlal Nehru Medical College, Datta Meghe Institute of Medical Sciences (Deemed to be University), Wardha, IND

**Keywords:** lightning injury, echocardiogram, cardiac biomarkers, electrocardiography, myocarditis

## Abstract

Lightning strikes are frequently encountered and are a major cause of morbidity and mortality. It could impair several organs, but the effects of electric current on the cardiovascular system contribute to the primary cause of cardiorespiratory arrest. These effects can be either transient or persistent, ranging from benign or life-threatening arrhythmias, ischemic injury, myocardial contusion, aortic injury, cardiomyopathy, and ventricular failure. Myocarditis has been an important but not very well-understood cause of cardiac dysfunction. Fulminant myocarditis is defined as patients presenting with severe heart failure, having a duration of <2 weeks of symptoms, and requiring inotropic or mechanical circulatory support. This condition can rapidly lead to hemodynamic instability and death. Resuscitation for a longer time increases the probability of favorable outcomes in young and previously healthy patients. This case report accounts for a case of a healthy young male who was struck by lightning while working on the farm and developed electrocardiographic changes along with positive cardiac biomarkers.

## Introduction

Lightning is a natural and unavoidable phenomenon. When the electrical potential difference between clouds and ground objects is more than 30,000 Volts, lightning strikes between the two can overcome the atmospheric electrical resistance and create currents ranging from 30,000 to 1,00,000 Amperes lasting between 0.001 and 0.1 seconds [1]. The temperature generated due to lightning is around 50,000°F, which is higher than the surface of the Sun, which is 10,000°F. Approximately one in four of the 2,00,000 to 3,00,000 lightning counts which occur annually, according to the Tornado and Storm Research Organization (TORRO), result in cloud-to-ground electrical discharges. It is estimated that 24,000 persons globally every year experience severe lightning-related injuries - the annual mortality rate from lightning range from 0.2 to 1.7 per million people around the globe. The body can be exposed to electrical currents in many ways, both directly and indirectly [2]. In a direct strike, which frequently affects the upper extremities, most of the current does not penetrate the victim; this is known as an "external flashover." Depending on the depth and intensity of the current penetration, effects could vary from mild erythema to severe multi-organ injury and cardiopulmonary arrest. The majority of injuries that happen inside are spurred on by indirect mechanisms of injury, such as the victim's contact with an electrifying object or the earth functioning as a conductor for lightning strikes nearby [3]. Lightning strikes can impact almost every organ system, and the prognosis is based on systemic complications. However, the most common and immediate cause of death from lightning is cardiac asystole and ventricular fibrillation, as it causes the depolarization of the whole myocardium [4]. The documented pathology includes cardiovascular effects, damaged central and peripheral nerve systems, severe muscle spasms with paralysis and necrosis, localized and deep burns, and skin modifications, including the recognizable fern-like or Lichtenberg figure erythema. The physical trauma may recover entirely or have long-term repercussions, such as psychological consequences for the survivors.

## Case presentation

**Figure 1:**
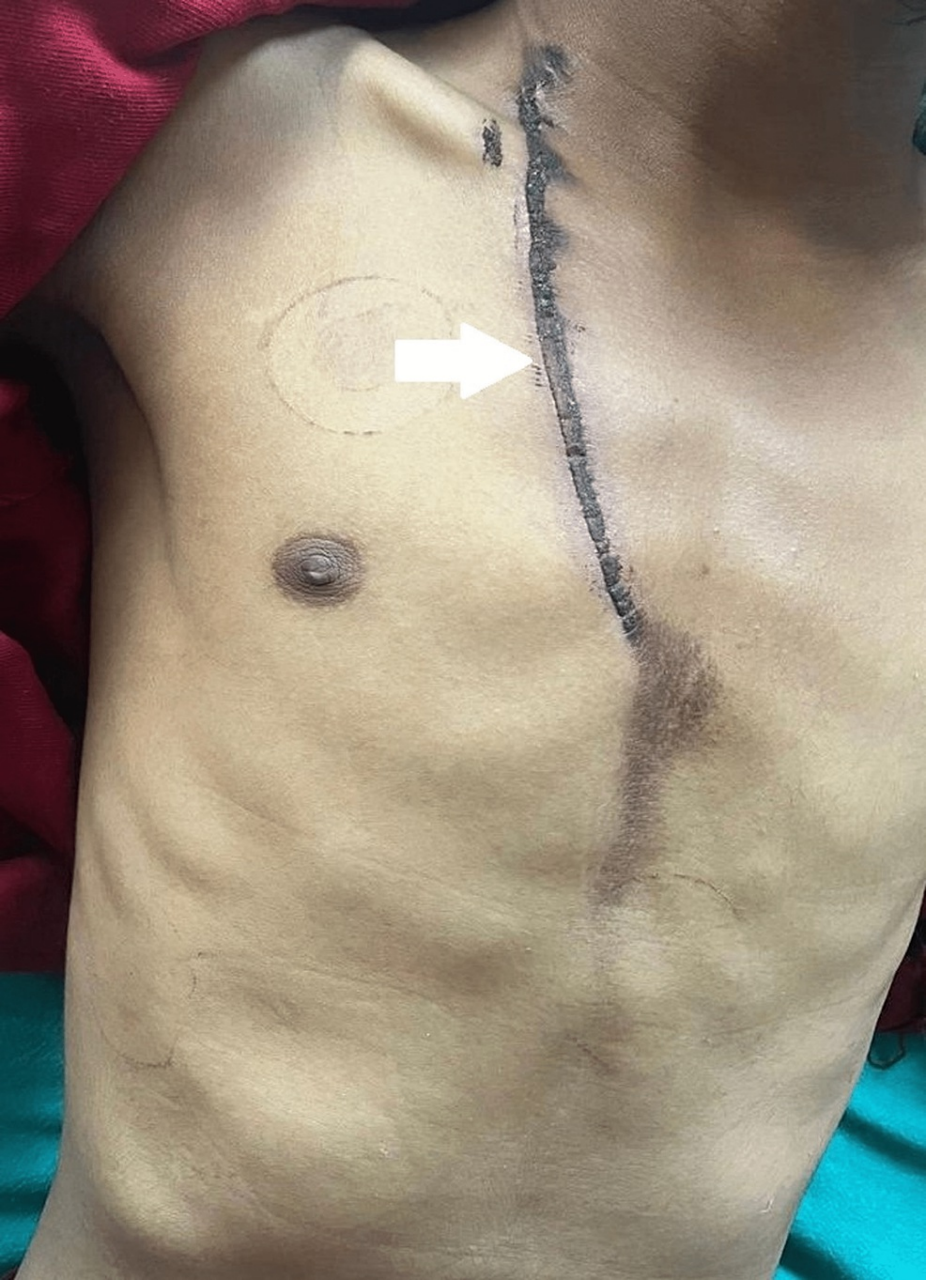
The superficial burn mark over the right chest (20 x 2 cm approximately) extends from the neck to the sternum.

**Figure 2:**
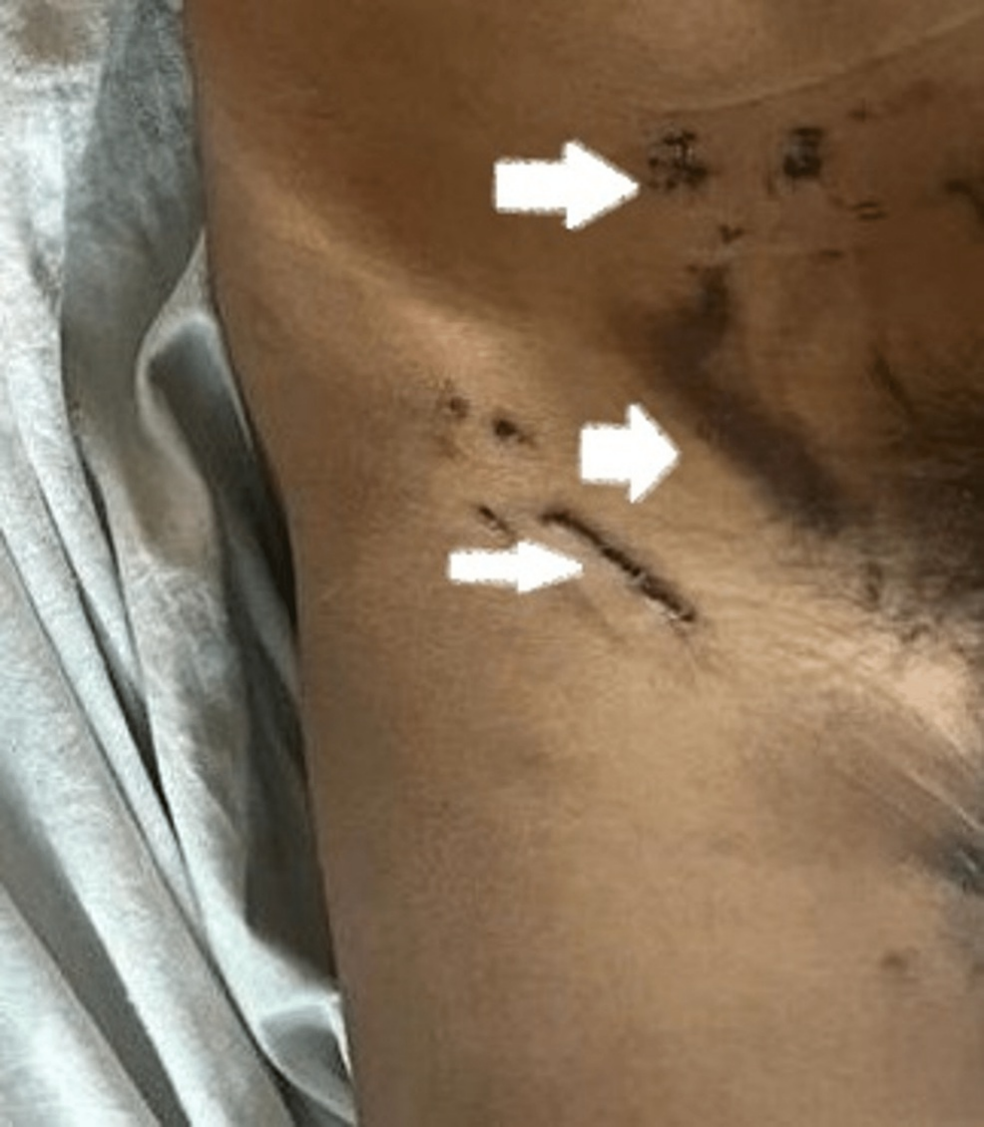
The superficial burn marks over the inguinal region and medial aspect of his right thigh.

During heavy rainfall and thunderstorm, an 18 years old male, while working on a farm in Devgaon (Amravati district in the Maharashtra state of India), was exposed to a lightning strike, following which he lost consciousness. The patient was brought to the emergency department of our hospital in a drowsy state. After regaining consciousness, he complained of severe chest pain radiating toward his left shoulder and left arm, associated with vomiting. He had no known past history of cardiac disease. On admission, his blood pressure was 100/70 mm Hg, with a regular heart rate of 76 beats per minute, respiratory rate of 18 cycles per minute, and axillary temperature of 36.5°C. There was no cyanosis or clubbing. The jugular venous pressure was normal. Local examination revealed superficial burn marks over the right side of the chest of approximately 20 x 2 cm extending from the neck to the sternum, along with a few superficial burn marks over his inguinal region and medial aspect of right thigh as lightning struck his body (Figures 1, 2).

**Figure 3:**
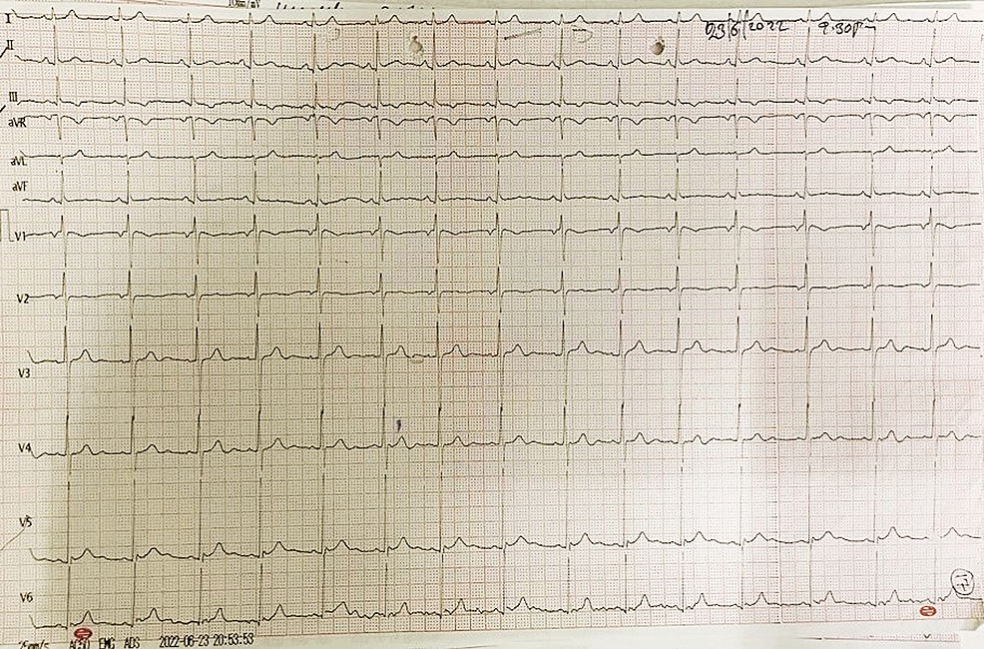
Electrocardiogram on admission showing sinus rhythm.

On cardiovascular system examination, precordium was normal in shape, apex beat was normal, S1 S2 were normal, and there was no murmur heard. Respiratory system examination did not reveal any abnormality. Abdominal examination was normal. On investigations, complete blood count, urea, creatinine, and electrolytes were within normal range, but serial Creatine Kinase-MB (CK-MB) levels showed a gradual rise. On admission, his CK-MB was 43 IU/L which rose to 45 IU/L after 6 hours (normal = 0-16 IU/L). His electrocardiogram on admission was normal, and his serial electrocardiograms (ECGs) were recorded (Figure 3).

**Figure 4:**
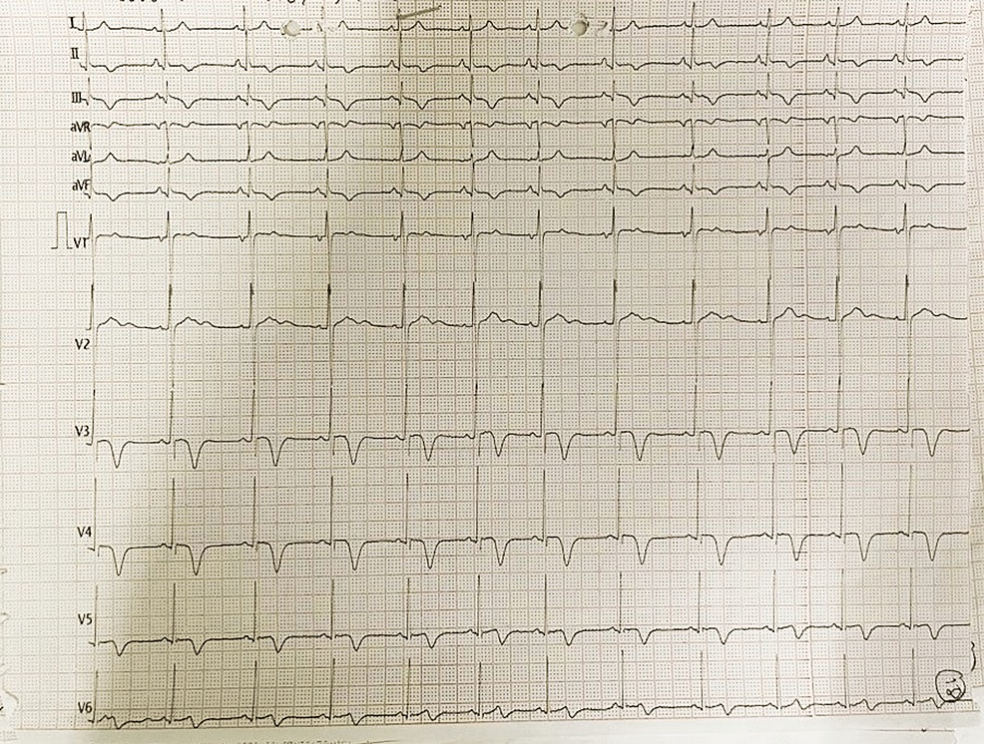
Electrocardiogram on day 3 of hospitalization showing T-wave inversion in leads II, III, aVF, and V3-6.

However, on the third day of hospitalization, his ECG showed T-wave inversion in leads II, III, aVF, and V3-6 (Figure 4).

He continued to have chest pain. There was no tenderness on palpation over the chest. There was no relation between respiration and chest pain. On auscultation pleural rub and pericardial rub were absent. His echocardiogram was suggestive of normal left ventricle (LV) function with a left ventricle ejection fraction (LVEF) of 65%. He was diagnosed to have lightning strike-induced myocarditis. He was started on antiemetics and intravenous (IV) fluids. During hospitalization, he did not develop any arrhythmia. After seven days, the patient had clinically improved, was hemodynamically stable, repeat echocardiogram that showed normal LV function with LVEF 65%, and was eventually discharged.

## Discussion

Anybody caught in a thunderstorm while involved in outdoor activities on exposed terrain should immediately look for a secure shelter. Immediately evacuate and remain distant from any body of water that could act as a peak or "beacon" for a possible strike. Find a grounded, enclosed building, or if that is not feasible, a metal-topped, enclosed vehicle (which will dissipate the electric current around the victim and directly to the ground) [1]. Hiding under a tree can be dangerous since a potential tree strike may cause an electric current to flow to the victim from the trunk, or vaporized sap could explode and cause serious injury. Furthermore, avoiding touching any insulated object from the ground after a strike is essential because it could cause it to store an electrical charge for a while. This does not apply to the victim's body, so if cardiopulmonary resuscitation, including defibrillation, is needed, it should be initiated immediately. According to NASA (National Aeronautics and Space Administration), at any given moment, more than 2,000 thunderstorms are active throughout the world producing 100 flashes per second [2-3]. Some 24,000 deaths, 240,000 injuries, and millions of dollars in property damage occur annually because of lightning. Lightning strikes are among the unusual causes of admission into the hospital [3]. Indirect injuries due to lightning may be through forest and house fires, blasts, and falling objects. A direct strike occurs when most of the current flow directly through the victim. Lightning can, directly and indirectly, affect cardiac rate and rhythm. The direct effect is through electrical and mechanical trauma, and an indirect effect by excessive catecholamine surge. Multiple ECG changes have been documented, ranging from specific and nonspecific ST segment changes and T-wave inversion to prolongation of QT interval, which is consistent with ischemia, contusions, pericarditis, repolarization abnormalities, and pericardial effusion [3-4]. ECG monitoring in apparently stable, lightning-injured patients can be of paramount importance. Patients may develop ventricular failure due to arrhythmias, cardiac ischemia, and myocardial necrosis. Autonomic stimulation and excessive catecholamine surge may result in Takotsubo syndrome with apical ballooning [4]. Myocarditis poses a challenge due to the diversity of clinical presentations. While a few patients experience benign, nonspecific symptoms, other patients develop symptoms that resemble myocardial ischemia. Another group of patients suffer hemodynamic collapse or abrupt death. Though transient, this condition should not be underestimated [4].

Our patient had intermittent chest pain, and serial cardiac enzymes estimation showed gradual elevation. The patient's serial ECG showed evolution from normal T waves to T wave inversion in leads II, III, aVF, and V3-6, leading us toward diagnosing lightning strike-induced myocarditis. During his hospital stay, no arrhythmia was recorded, and even the QTc interval was normal.

## Conclusions

Early diagnosis of myocarditis is important since these patients are at risk of rapidly deteriorating and a relatively high proportion will need mechanical circulatory support. ‍Significant cardiac and non-cardiac problems can arise from lightning strikes, resulting in devastating outcomes. The case fatality rate has declined during the past three decades. This can be linked to various factors, such as improved public awareness of safety standards for outdoor and indoor activities, lightning protection systems in buildings and other structures, and medical procedures and protocols to manage victims immediately and correctly. The consequences of lightning strikes on the heart are a prominent cause of death and morbidity. Detailed history, physical examination, 12-lead ECG, cardiac biomarkers, prolonged electrocardiographic monitoring (for ventricular arrhythmias), and assessment for signs and symptoms of hemodynamic compromise should also be included in the evaluation. Cardiac magnetic resonance imaging (MRI) and/or endomyocardial biopsy are usually the most highly specific investigations, and they can help in establishing the diagnosis.
